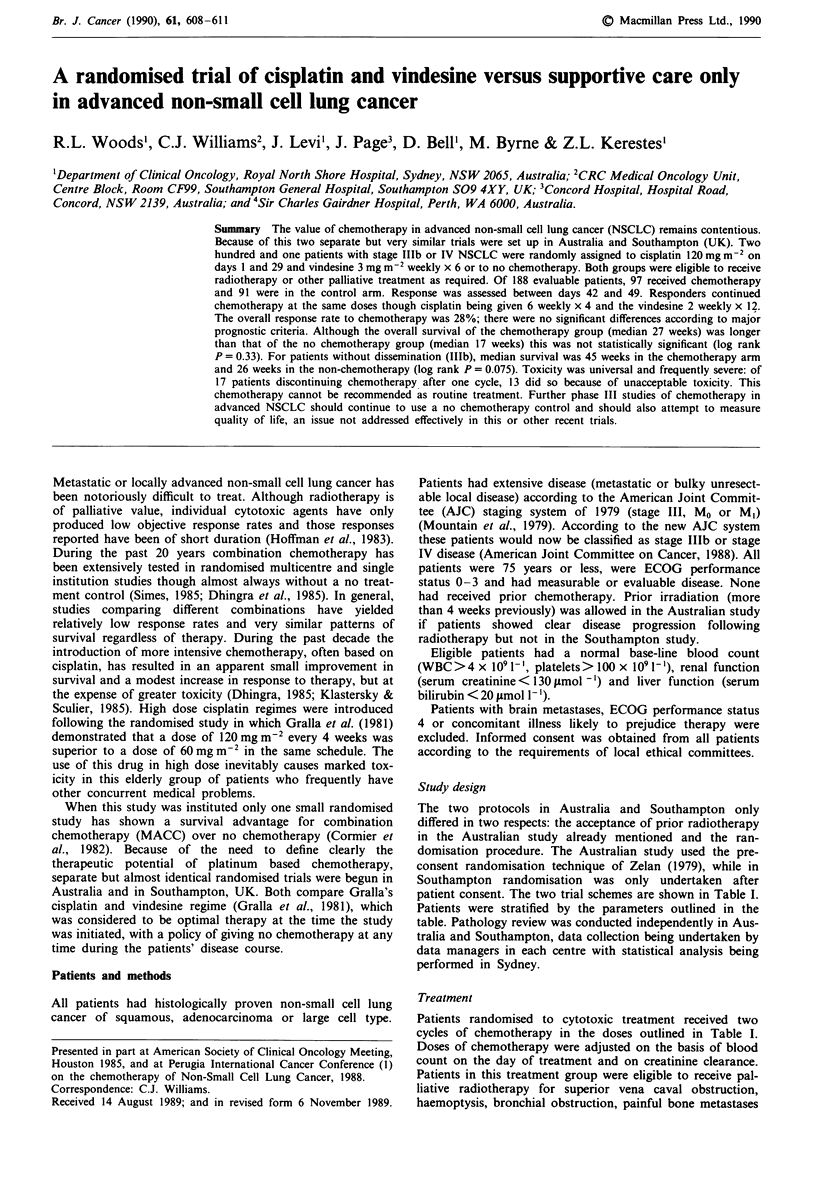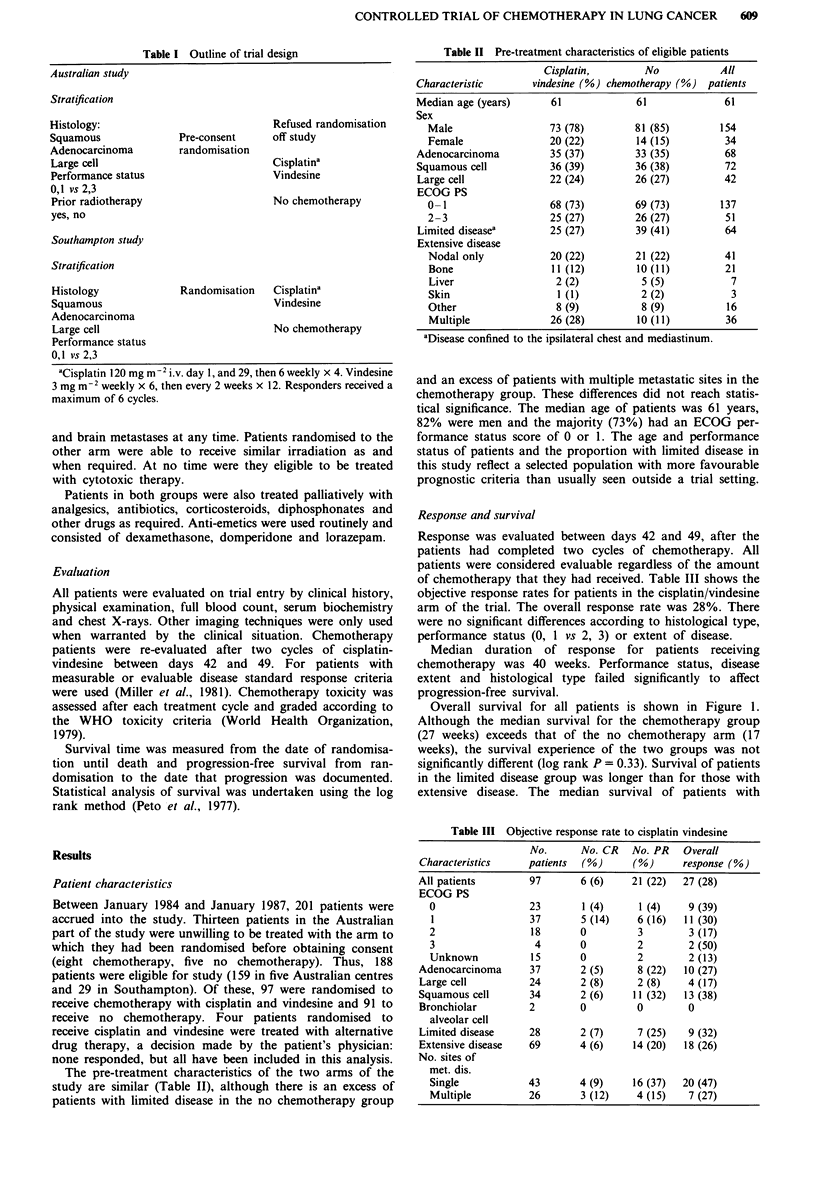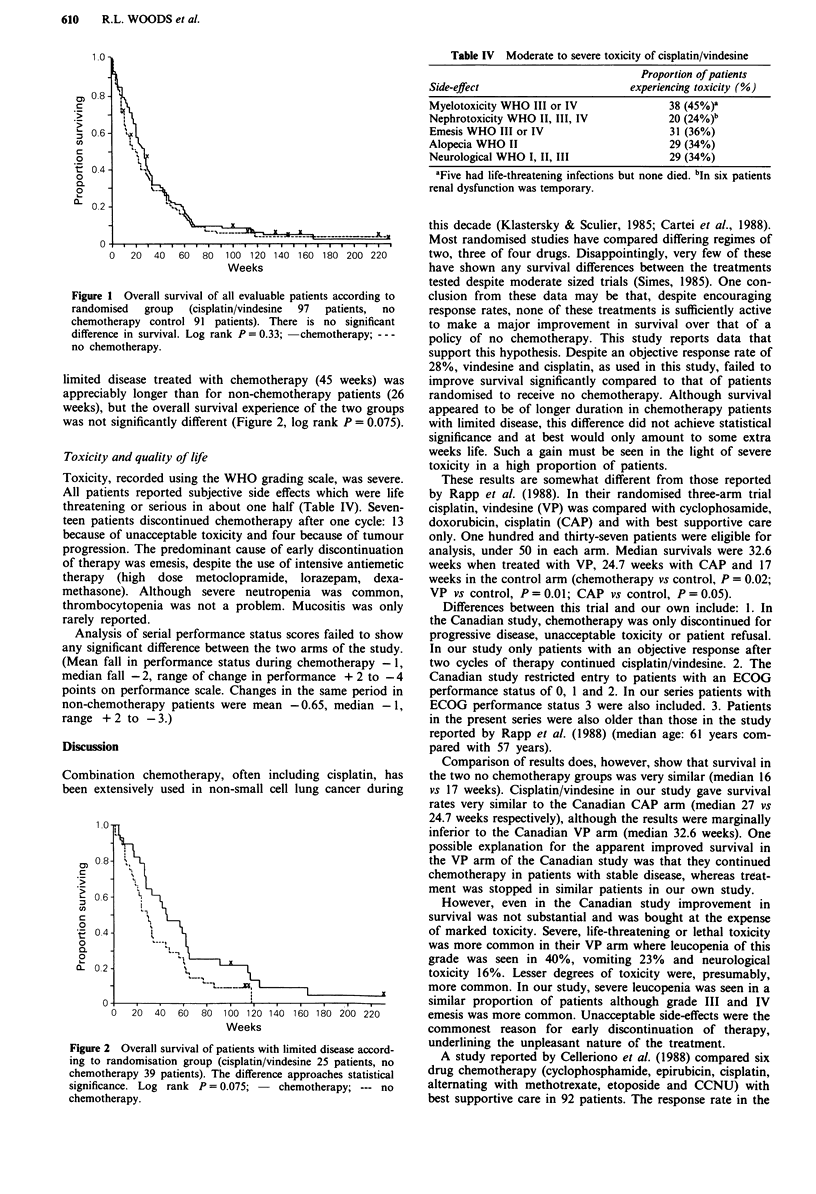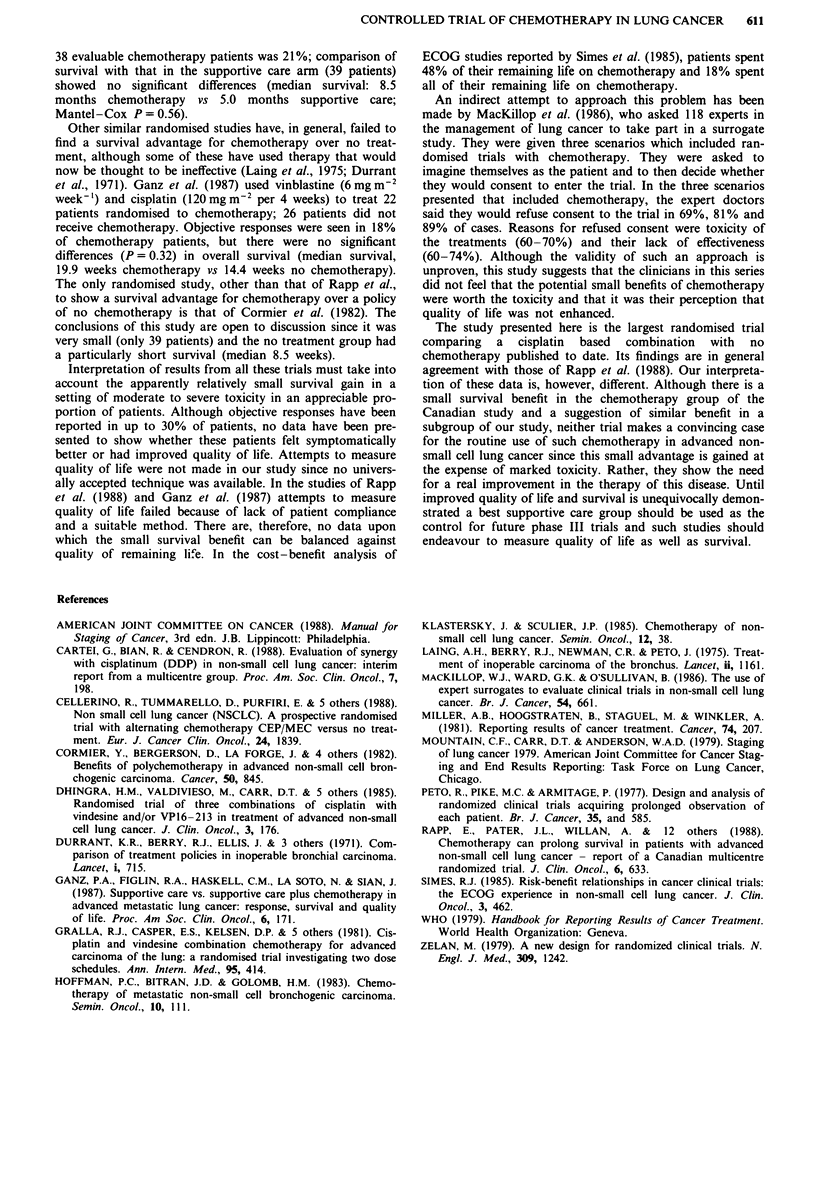# A randomised trial of cisplatin and vindesine versus supportive care only in advanced non-small cell lung cancer.

**DOI:** 10.1038/bjc.1990.135

**Published:** 1990-04

**Authors:** R. L. Woods, C. J. Williams, J. Levi, J. Page, D. Bell, M. Byrne, Z. L. Kerestes

**Affiliations:** Department of Clinical Oncology, Royal North Shore Hospital, Sydney, NSW, Australia.

## Abstract

The value of chemotherapy in advanced non-small cell lung cancer (NSCLC) remains contentious. Because of this two separate but very similar trials were set up in Australia and Southampton (UK). Two hundred and one patients with stage IIIb or IV NSCLC were randomly assigned to cisplatin 120 mg m-2 on days 1 and 29 and vindesine 3 mg m-2 weekly x 6 or to no chemotherapy. Both groups were eligible to receive radiotherapy or other palliative treatment as required. Of 188 evaluable patients, 97 received chemotherapy and 91 were in the control arm. Response was assessed between days 42 and 49. Responders continued chemotherapy at the same doses though cisplatin being given 6 weekly x 4 and the vindesine 2 weekly x 12. The overall response rate to chemotherapy was 28%; there were no significant differences according to major prognostic criteria. Although the overall survival of the chemotherapy group (median 27 weeks) was longer than that of the no chemotherapy group (median 17 weeks) this was not statistically significant (log rank P = 0.33). For patients without dissemination (IIIb), median survival was 45 weeks in the chemotherapy arm and 26 weeks in the non-chemotherapy (log rank P = 0.075). Toxicity was universal and frequently severe: of 17 patients discontinuing chemotherapy after one cycle, 13 did so because of unacceptable toxicity. This chemotherapy cannot be recommended as routine treatment. Further phase III studies of chemotherapy in advanced NSCLC should continue to use a no chemotherapy control and should also attempt to measure quality of life, an issue not addressed effectively in this or other recent trials.


Br. J. Cancer (1990), 61, 608-611 © Macmillan Press Ltd., 1990

### A randomised trial of cisplatin and vindesine versus supportive care only in advanced non-small cell lung cancer

R.L. Woods[1], C.J. Williams[2], J. Levi[1], J. Page[3], D. Bell[1], M. Byrne & Z.L. Kerestes[1]

[1]*Department of Clinical Oncology, Royal North Shore Hospital, Sydney, NSW 2065, Australia;* [2]*CRC Medical Oncology Unit, Centre Block, Room CF99, Southampton General Hospital, Southampton SO9 4XY, UK;* [3]*Concord Hospital, Hospital Road, Concord, NSW 2139, Australia; and* [4]*Sir Charles Gairdner Hospital, Perth, WA 6000, Australia.*

**Summary** The value of chemotherapy in advanced non-small cell lung cancer (NSCLC) remains contentious. Because of this two separate but very similar trials were set up in Australia and Southampton (UK). Two hundred and one patients with stage IIIb or IV NSCLC were randomly assigned to cisplatin 120 mg $m^{-2}$ on days 1 and 29 and vindesine 3 mg $m^{-2}$ weekly × 6 or to no chemotherapy. Both groups were eligible to receive radiotherapy or other palliative treatment as required. Of 188 evaluable patients, 97 received chemotherapy and 91 were in the control arm. Response was assessed between days 42 and 49. Responders continued chemotherapy at the same doses though cisplatin being given 6 weekly × 4 and the vindesine 2 weekly × 12. The overall response rate to chemotherapy was 28%; there were no significant differences according to major prognostic criteria. Although the overall survival of the chemotherapy group (median 27 weeks) was longer than that of the no chemotherapy group (median 17 weeks) this was not statistically significant (log rank $P = 0.33$). For patients without dissemination (IIIb), median survival was 45 weeks in the chemotherapy arm and 26 weeks in the non-chemotherapy (log rank $P = 0.075$). Toxicity was universal and frequently severe: of 17 patients discontinuing chemotherapy after one cycle, 13 did so because of unacceptable toxicity. This chemotherapy cannot be recommended as routine treatment. Further phase III studies of chemotherapy in advanced NSCLC should continue to use a no chemotherapy control and should also attempt to measure quality of life, an issue not addressed effectively in this or other recent trials.

Metastatic or locally advanced non-small cell lung cancer has been notoriously difficult to treat. Although radiotherapy is of palliative value, individual cytotoxic agents have only produced low objective response rates and those responses reported have been of short duration (Hoffman *et al.*, 1983). During the past 20 years combination chemotherapy has been extensively tested in randomised multicentre and single institution studies though almost always without a no treatment control (Simes, 1985; Dhingra *et al.*, 1985). In general, studies comparing different combinations have yielded relatively low response rates and very similar patterns of survival regardless of therapy. During the past decade the introduction of more intensive chemotherapy, often based on cisplatin, has resulted in an apparent small improvement in survival and a modest increase in response to therapy, but at the expense of greater toxicity (Dhingra, 1985; Klastersky & Sculier, 1985). High dose cisplatin regimes were introduced following the randomised study in which Gralla *et al.* (1981) demonstrated that a dose of 120 mg $m^{-2}$ every 4 weeks was superior to a dose of 60 mg $m^{-2}$ in the same schedule. The use of this drug in high dose inevitably causes marked toxicity in this elderly group of patients who frequently have other concurrent medical problems.

When this study was instituted only one small randomised study has shown a survival advantage for combination chemotherapy (MACC) over no chemotherapy (Cormier *et al.*, 1982). Because of the need to define clearly the therapeutic potential of platinum based chemotherapy, separate but almost identical randomised trials were begun in Australia and in Southampton, UK. Both compare Gralla's cisplatin and vindesine regime (Gralla *et al.*, 1981), which was considered to be optimal therapy at the time the study was initiated, with a policy of giving no chemotherapy at any time during the patients' disease course.

### Patients and methods

All patients had histologically proven non-small cell lung cancer of squamous, adenocarcinoma or large cell type. Patients had extensive disease (metastatic or bulky unresectable local disease) according to the American Joint Committee (AJC) staging system of 1979 (stage III, $M_0$ or $M_1$) (Mountain *et al.*, 1979). According to the new AJC system these patients would now be classified as stage IIIb or stage IV disease (American Joint Committee on Cancer, 1988). All patients were 75 years or less, were ECOG performance status 0–3 and had measurable or evaluable disease. None had received prior chemotherapy. Prior irradiation (more than 4 weeks previously) was allowed in the Australian study if patients showed clear disease progression following radiotherapy but not in the Southampton study.

Eligible patients had a normal base-line blood count (WBC $> 4 \times 10^9 l^{-1}$, platelets $> 100 \times 10^9 l^{-1}$), renal function (serum creatinine $< 130 \mu mol^{-1}$) and liver function (serum bilirubin $< 20 \mu mol\, l^{-1}$).

Patients with brain metastases, ECOG performance status 4 or concomitant illness likely to prejudice therapy were excluded. Informed consent was obtained from all patients according to the requirements of local ethical committees.

### *Study design*

The two protocols in Australia and Southampton only differed in two respects: the acceptance of prior radiotherapy in the Australian study already mentioned and the randomisation procedure. The Australian study used the preconsent randomisation technique of Zelan (1979), while in Southampton randomisation was only undertaken after patient consent. The two trial schemes are shown in Table I. Patients were stratified by the parameters outlined in the table. Pathology review was conducted independently in Australia and Southampton, data collection being undertaken by data managers in each centre with statistical analysis being performed in Sydney.

### *Treatment*

Patients randomised to cytotoxic treatment received two cycles of chemotherapy in the doses outlined in Table I. Doses of chemotherapy were adjusted on the basis of blood count on the day of treatment and on creatinine clearance. Patients in this treatment group were eligible to receive palliative radiotherapy for superior vena caval obstruction, haemoptysis, bronchial obstruction, painful bone metastases

Presented in part at American Society of Clinical Oncology Meeting, Houston 1985, and at Perugia International Cancer Conference (1) on the chemotherapy of Non-Small Cell Lung Cancer, 1988.
Correspondence: C.J. Williams.
Received 14 August 1989; and in revised form 6 November 1989.

**Table I** Outline of trial design

| | | |
|---|---|---|
| *Australian study* | | |
| *Stratification* | | |
| Histology: | | Refused randomisation |
| Squamous | Pre-consent | off study |
| Adenocarcinoma | randomisation | |
| Large cell | | Cisplatin[a] |
| Performance status | | Vindesine |
| 0,1 *vs* 2,3 | | |
| Prior radiotherapy | | No chemotherapy |
| yes, no | | |
| *Southampton study* | | |
| *Stratification* | | |
| Histology | Randomisation | Cisplatin[a] |
| Squamous | | Vindesine |
| Adenocarcinoma | | |
| Large cell | | No chemotherapy |
| Performance status | | |
| 0,1 *vs* 2,3 | | |

[a]Cisplatin 120 mg $m^{-2}$ i.v. day 1, and 29, then 6 weekly × 4. Vindesine 3 mg $m^{-2}$ weekly × 6, then every 2 weeks × 12. Responders received a maximum of 6 cycles.

and brain metastases at any time. Patients randomised to the other arm were able to receive similar irradiation as and when required. At no time were they eligible to be treated with cytotoxic therapy.

Patients in both groups were also treated palliatively with analgesics, antibiotics, corticosteroids, diphosphonates and other drugs as required. Anti-emetics were used routinely and consisted of dexamethasone, domperidone and lorazepam.

### *Evaluation*

All patients were evaluated on trial entry by clinical history, physical examination, full blood count, serum biochemistry and chest X-rays. Other imaging techniques were only used when warranted by the clinical situation. Chemotherapy patients were re-evaluated after two cycles of cisplatin-vindesine between days 42 and 49. For patients with measurable or evaluable disease standard response criteria were used (Miller *et al.*, 1981). Chemotherapy toxicity was assessed after each treatment cycle and graded according to the WHO toxicity criteria (World Health Organization, 1979).

Survival time was measured from the date of randomisation until death and progression-free survival from randomisation to the date that progression was documented. Statistical analysis of survival was undertaken using the log rank method (Peto *et al.*, 1977).

### Results

### *Patient characteristics*

Between January 1984 and January 1987, 201 patients were accrued into the study. Thirteen patients in the Australian part of the study were unwilling to be treated with the arm to which they had been randomised before obtaining consent (eight chemotherapy, five no chemotherapy). Thus, 188 patients were eligible for study (159 in five Australian centres and 29 in Southampton). Of these, 97 were randomised to receive chemotherapy with cisplatin and vindesine and 91 to receive no chemotherapy. Four patients randomised to receive cisplatin and vindesine were treated with alternative drug therapy, a decision made by the patient's physician: none responded, but all have been included in this analysis.

The pre-treatment characteristics of the two arms of the study are similar (Table II), although there is an excess of patients with limited disease in the no chemotherapy group

**Table II** Pre-treatment characteristics of eligible patients

| *Characteristic* | *Cisplatin, vindesine (%)* | *No chemotherapy (%)* | *All patients* |
|---|---|---|---|
| Median age (years) | 61 | 61 | 61 |
| Sex | | | |
| Male | 73 (78) | 81 (85) | 154 |
| Female | 20 (22) | 14 (15) | 34 |
| Adenocarcinoma | 35 (37) | 33 (35) | 68 |
| Squamous cell | 36 (39) | 36 (38) | 72 |
| Large cell | 22 (24) | 26 (27) | 42 |
| ECOG PS | | | |
| 0–1 | 68 (73) | 69 (73) | 137 |
| 2–3 | 25 (27) | 26 (27) | 51 |
| Limited disease[a] | 25 (27) | 39 (41) | 64 |
| Extensive disease | | | |
| Nodal only | 20 (22) | 21 (22) | 41 |
| Bone | 11 (12) | 10 (11) | 21 |
| Liver | 2 (2) | 5 (5) | 7 |
| Skin | 1 (1) | 2 (2) | 3 |
| Other | 8 (9) | 8 (9) | 16 |
| Multiple | 26 (28) | 10 (11) | 36 |

[a]Disease confined to the ipsilateral chest and mediastinum.

and an excess of patients with multiple metastatic sites in the chemotherapy group. These differences did not reach statistical significance. The median age of patients was 61 years, 82% were men and the majority (73%) had an ECOG performance status score of 0 or 1. The age and performance status of patients and the proportion with limited disease in this study reflect a selected population with more favourable prognostic criteria than usually seen outside a trial setting.

### *Response and survival*

Response was evaluated between days 42 and 49, after the patients had completed two cycles of chemotherapy. All patients were considered evaluable regardless of the amount of chemotherapy that they had received. Table III shows the objective response rates for patients in the cisplatin/vindesine arm of the trial. The overall response rate was 28%. There were no significant differences according to histological type, performance status (0, 1 *vs* 2, 3) or extent of disease.

Median duration of response for patients receiving chemotherapy was 40 weeks. Performance status, disease extent and histological type failed significantly to affect progression-free survival.

Overall survival for all patients is shown in Figure 1. Although the median survival for the chemotherapy group (27 weeks) exceeds that of the no chemotherapy arm (17 weeks), the survival experience of the two groups was not significantly different (log rank $P = 0.33$). Survival of patients in the limited disease group was longer than for those with extensive disease. The median survival of patients with

**Table III** Objective response rate to cisplatin vindesine

| *Characteristics* | *No. patients* | *No. CR (%)* | *No. PR (%)* | *Overall response (%)* |
|---|---|---|---|---|
| All patients | 97 | 6 (6) | 21 (22) | 27 (28) |
| ECOG PS | | | | |
| 0 | 23 | 1 (4) | 1 (4) | 9 (39) |
| 1 | 37 | 5 (14) | 6 (16) | 11 (30) |
| 2 | 18 | 0 | 3 | 3 (17) |
| 3 | 4 | 0 | 2 | 2 (50) |
| Unknown | 15 | 0 | 2 | 2 (13) |
| Adenocarcinoma | 37 | 2 (5) | 8 (22) | 10 (27) |
| Large cell | 24 | 2 (8) | 2 (8) | 4 (17) |
| Squamous cell | 34 | 2 (6) | 11 (32) | 13 (38) |
| Bronchiolar alveolar cell | 2 | 0 | 0 | 0 |
| Limited disease | 28 | 2 (7) | 7 (25) | 9 (32) |
| Extensive disease | 69 | 4 (6) | 14 (20) | 18 (26) |
| No. sites of met. dis. | | | | |
| Single | 43 | 4 (9) | 16 (37) | 20 (47) |
| Multiple | 26 | 3 (12) | 4 (15) | 7 (27) |

**Figure 1** Overall survival of all evaluable patients according to randomised group (cisplatin/vindesine 97 patients, no chemotherapy control 91 patients). There is no significant difference in survival. Log rank $P = 0.33$; —chemotherapy; --- no chemotherapy.

limited disease treated with chemotherapy (45 weeks) was appreciably longer than for non-chemotherapy patients (26 weeks), but the overall survival experience of the two groups was not significantly different (Figure 2, log rank $P = 0.075$).

*Toxicity and quality of life*

Toxicity, recorded using the WHO grading scale, was severe. All patients reported subjective side effects which were life threatening or serious in about one half (Table IV). Seventeen patients discontinued chemotherapy after one cycle: 13 because of unacceptable toxicity and four because of tumour progression. The predominant cause of early discontinuation of therapy was emesis, despite the use of intensive antiemetic therapy (high dose metoclopramide, lorazepam, dexamethasone). Although severe neutropenia was common, thrombocytopenia was not a problem. Mucositis was only rarely reported.

Analysis of serial performance status scores failed to show any significant difference between the two arms of the study. (Mean fall in performance status during chemotherapy −1, median fall −2, range of change in performance +2 to −4 points on performance scale. Changes in the same period in non-chemotherapy patients were mean −0.65, median −1, range +2 to −3.)

### Discussion

Combination chemotherapy, often including cisplatin, has been extensively used in non-small cell lung cancer during

**Figure 2** Overall survival of patients with limited disease according to randomisation group (cisplatin/vindesine 25 patients, no chemotherapy 39 patients). The difference approaches statistical significance. Log rank $P = 0.075$; — chemotherapy; --- no chemotherapy.

**Table IV** Moderate to severe toxicity of cisplatin/vindesine

| *Side-effect* | *Proportion of patients experiencing toxicity (%)* |
|---|---|
| Myelotoxicity WHO III or IV | 38 (45%)[a] |
| Nephrotoxicity WHO II, III, IV | 20 (24%)[b] |
| Emesis WHO III or IV | 31 (36%) |
| Alopecia WHO II | 29 (34%) |
| Neurological WHO I, II, III | 29 (34%) |

[a]Five had life-threatening infections but none died. [b]In six patients renal dysfunction was temporary.

this decade (Klastersky & Sculier, 1985; Cartei *et al.*, 1988). Most randomised studies have compared differing regimes of two, three of four drugs. Disappointingly, very few of these have shown any survival differences between the treatments tested despite moderate sized trials (Simes, 1985). One conclusion from these data may be that, despite encouraging response rates, none of these treatments is sufficiently active to make a major improvement in survival over that of a policy of no chemotherapy. This study reports data that support this hypothesis. Despite an objective response rate of 28%, vindesine and cisplatin, as used in this study, failed to improve survival significantly compared to that of patients randomised to receive no chemotherapy. Although survival appeared to be of longer duration in chemotherapy patients with limited disease, this difference did not achieve statistical significance and at best would only amount to some extra weeks life. Such a gain must be seen in the light of severe toxicity in a high proportion of patients.

These results are somewhat different from those reported by Rapp *et al.* (1988). In their randomised three-arm trial cisplatin, vindesine (VP) was compared with cyclophosamide, doxorubicin, cisplatin (CAP) and with best supportive care only. One hundred and thirty-seven patients were eligible for analysis, under 50 in each arm. Median survivals were 32.6 weeks when treated with VP, 24.7 weeks with CAP and 17 weeks in the control arm (chemotherapy *vs* control, $P = 0.02$; VP *vs* control, $P = 0.01$; CAP *vs* control, $P = 0.05$).

Differences between this trial and our own include: 1. In the Canadian study, chemotherapy was only discontinued for progressive disease, unacceptable toxicity or patient refusal. In our study only patients with an objective response after two cycles of therapy continued cisplatin/vindesine. 2. The Canadian study restricted entry to patients with an ECOG performance status of 0, 1 and 2. In our series patients with ECOG performance status 3 were also included. 3. Patients in the present series were also older than those in the study reported by Rapp *et al.* (1988) (median age: 61 years compared with 57 years).

Comparison of results does, however, show that survival in the two no chemotherapy groups was very similar (median 16 *vs* 17 weeks). Cisplatin/vindesine in our study gave survival rates very similar to the Canadian CAP arm (median 27 *vs* 24.7 weeks respectively), although the results were marginally inferior to the Canadian VP arm (median 32.6 weeks). One possible explanation for the apparent improved survival in the VP arm of the Canadian study was that they continued chemotherapy in patients with stable disease, whereas treatment was stopped in similar patients in our own study.

However, even in the Canadian study improvement in survival was not substantial and was bought at the expense of marked toxicity. Severe, life-threatening or lethal toxicity was more common in their VP arm where leucopenia of this grade was seen in 40%, vomiting 23% and neurological toxicity 16%. Lesser degrees of toxicity were, presumably, more common. In our study, severe leucopenia was seen in a similar proportion of patients although grade III and IV emesis was more common. Unacceptable side-effects were the commonest reason for early discontinuation of therapy, underlining the unpleasant nature of the treatment.

A study reported by Celleriono *et al.* (1988) compared six drug chemotherapy (cyclophosphamide, epirubicin, cisplatin, alternating with methotrexate, etoposide and CCNU) with best supportive care in 92 patients. The response rate in the

38 evaluable chemotherapy patients was 21%; comparison of survival with that in the supportive care arm (39 patients) showed no significant differences (median survival: 8.5 months chemotherapy *vs* 5.0 months supportive care; Mantel–Cox $P = 0.56$).

Other similar randomised studies have, in general, failed to find a survival advantage for chemotherapy over no treatment, although some of these have used therapy that would now be thought to be ineffective (Laing *et al.*, 1975; Durrant *et al.*, 1971). Ganz *et al.* (1987) used vinblastine ($6\ mg\ m^{-2}$ $week^{-1}$) and cisplatin ($120\ mg\ m^{-2}$ per 4 weeks) to treat 22 patients randomised to chemotherapy; 26 patients did not receive chemotherapy. Objective responses were seen in 18% of chemotherapy patients, but there were no significant differences ($P = 0.32$) in overall survival (median survival, 19.9 weeks chemotherapy *vs* 14.4 weeks no chemotherapy). The only randomised study, other than that of Rapp *et al.*, to show a survival advantage for chemotherapy over a policy of no chemotherapy is that of Cormier *et al.* (1982). The conclusions of this study are open to discussion since it was very small (only 39 patients) and the no treatment group had a particularly short survival (median 8.5 weeks).

Interpretation of results from all these trials must take into account the apparently relatively small survival gain in a setting of moderate to severe toxicity in an appreciable proportion of patients. Although objective responses have been reported in up to 30% of patients, no data have been presented to show whether these patients felt symptomatically better or had improved quality of life. Attempts to measure quality of life were not made in our study since no universally accepted technique was available. In the studies of Rapp *et al.* (1988) and Ganz *et al.* (1987) attempts to measure quality of life failed because of lack of patient compliance and a suitable method. There are, therefore, no data upon which the small survival benefit can be balanced against quality of remaining life. In the cost–benefit analysis of ECOG studies reported by Simes *et al.* (1985), patients spent 48% of their remaining life on chemotherapy and 18% spent all of their remaining life on chemotherapy.

An indirect attempt to approach this problem has been made by MacKillop *et al.* (1986), who asked 118 experts in the management of lung cancer to take part in a surrogate study. They were given three scenarios which included randomised trials with chemotherapy. They were asked to imagine themselves as the patient and to then decide whether they would consent to enter the trial. In the three scenarios presented that included chemotherapy, the expert doctors said they would refuse consent to the trial in 69%, 81% and 89% of cases. Reasons for refused consent were toxicity of the treatments (60–70%) and their lack of effectiveness (60–74%). Although the validity of such an approach is unproven, this study suggests that the clinicians in this series did not feel that the potential small benefits of chemotherapy were worth the toxicity and that it was their perception that quality of life was not enhanced.

The study presented here is the largest randomised trial comparing a cisplatin based combination with no chemotherapy published to date. Its findings are in general agreement with those of Rapp *et al.* (1988). Our interpretation of these data is, however, different. Although there is a small survival benefit in the chemotherapy group of the Canadian study and a suggestion of similar benefit in a subgroup of our study, neither trial makes a convincing case for the routine use of such chemotherapy in advanced non-small cell lung cancer since this small advantage is gained at the expense of marked toxicity. Rather, they show the need for a real improvement in the therapy of this disease. Until improved quality of life and survival is unequivocally demonstrated a best supportive care group should be used as the control for future phase III trials and such studies should endeavour to measure quality of life as well as survival.

### References

AMERICAN JOINT COMMITTEE ON CANCER (1988). *Manual for Staging of Cancer*, 3rd edn. J.B. Lippincott: Philadelphia.

CARTEI, G., BIAN, R. & CENDRON, R. (1988). Evaluation of synergy with cisplatinum (DDP) in non-small cell lung cancer: interim report from a multicentre group. *Proc. Am. Soc. Clin. Oncol.*, **7**, 198.

CELLERINO, R., TUMMARELLO, D., PURFIRI, E. & 5 others (1988). Non small cell lung cancer (NSCLC). A prospective randomised trial with alternating chemotherapy CEP/MEC versus no treatment. *Eur. J. Cancer Clin. Oncol.*, **24**, 1839.

CORMIER, Y., BERGERSON, D., LA FORGE, J. & 4 others (1982). Benefits of polychemotherapy in advanced non-small cell bronchogenic carcinoma. *Cancer*, **50**, 845.

DHINGRA, H.M., VALDIVIESO, M., CARR, D.T. & 5 others (1985). Randomised trial of three combinations of cisplatin with vindesine and/or VP16–213 in treatment of advanced non-small cell lung cancer. *J. Clin. Oncol.*, **3**, 176.

DURRANT, K.R., BERRY, R.J., ELLIS, J. & 3 others (1971). Comparison of treatment policies in inoperable bronchial carcinoma. *Lancet*, **i**, 715.

GANZ, P.A., FIGLIN, R.A., HASKELL, C.M., LA SOTO, N. & SIAN, J. (1987). Supportive care vs. supportive care plus chemotherapy in advanced metastatic lung cancer: response, survival and quality of life. *Proc. Am Soc. Clin. Oncol.*, **6**, 171.

GRALLA, R.J., CASPER, E.S., KELSEN, D.P. & 5 others (1981). Cisplatin and vindesine combination chemotherapy for advanced carcinoma of the lung: a randomised trial investigating two dose schedules. *Ann. Intern. Med.*, **95**, 414.

HOFFMAN, P.C., BITRAN, J.D. & GOLOMB, H.M. (1983). Chemotherapy of metastatic non-small cell bronchogenic carcinoma. *Semin. Oncol.*, **10**, 111.

KLASTERSKY, J. & SCULIER, J.P. (1985). Chemotherapy of non-small cell lung cancer. *Semin. Oncol.*, **12**, 38.

LAING, A.H., BERRY, R.J., NEWMAN, C.R. & PETO, J. (1975). Treatment of inoperable carcinoma of the bronchus. *Lancet*, **ii**, 1161.

MACKILLOP, W.J., WARD, G.K. & O'SULLIVAN, B. (1986). The use of expert surrogates to evaluate clinical trials in non-small cell lung cancer. *Br. J. Cancer*, **54**, 661.

MILLER, A.B., HOOGSTRATEN, B., STAGUEL, M. & WINKLER, A. (1981). Reporting results of cancer treatment. *Cancer*, **74**, 207.

MOUNTAIN, C.F., CARR, D.T. & ANDERSON, W.A.D. (1979). Staging of lung cancer 1979. American Joint Committee for Cancer Staging and End Results Reporting: Task Force on Lung Cancer, Chicago.

PETO, R., PIKE, M.C. & ARMITAGE, P. (1977). Design and analysis of randomized clinical trials acquiring prolonged observation of each patient. *Br. J. Cancer*, **35**, and 585.

RAPP, E., PATER, J.L., WILLAN, A. & 12 others (1988). Chemotherapy can prolong survival in patients with advanced non-small cell lung cancer – report of a Canadian multicentre randomized trial. *J. Clin. Oncol.*, **6**, 633.

SIMES, R.J. (1985). Risk-benefit relationships in cancer clinical trials: the ECOG experience in non-small cell lung cancer. *J. Clin. Oncol.*, **3**, 462.

WHO (1979). *Handbook for Reporting Results of Cancer Treatment*. World Health Organization: Geneva.

ZELAN, M. (1979). A new design for randomized clinical trials. *N. Engl. J. Med.*, **309**, 1242.